# Programmable Functional Silicification of DNA Origami Nanostructures

**DOI:** 10.1002/adma.74516

**Published:** 2026-08-05

**Authors:** Anna V. Baptist, Lasse Guericke, Philipp Mauker, Oliver Thorn‐Seshold, Amelie Heuer‐Jungemann

**Affiliations:** ^1^ Faculty of Chemistry and Chemical Biology TU Dortmund and Research Center One Health Ruhr Dortmund Germany; ^2^ Max Planck Institute of Biochemistry, and Center for Nanoscience LMU Munich Munich Germany; ^3^ Faculty of Chemistry and Food Chemistry Dresden University of Technology Dresden Germany

**Keywords:** cellular uptake, dissolvability, DNA origami, fluorescence, silicification

## Abstract

The silicification of DNA origami nanostructures offers a powerful strategy for enhancing their mechanical stability and resistance against detrimental environmental conditions. In the past years, several studies have investigated key aspects of the silicification process, resulting in a variety of established protocols. However, until now, the silica coating generally served as a passive protective layer or as the base for the further deposition of inorganic materials, but it did not carry any additional functionality itself. Here, we introduce two complementary, programmable approaches for the direct fabrication of functionalized silica coatings of DNA origami nanostructures. First, we synthesized a fluorescein‐bearing silica precursor which imparts fluorescence to the silica coating of both individual DNA origami nanostructures and crystals, enabling intracellular tracking of silica‐stabilized structures. Second, we employed a silica precursor containing a disulfide bridge to generate a redox responsive silica coating that degrades in a reducing environment. By introducing functionality at the precursor level, our approach establishes silicification as a modular platform for constructing responsive and traceable DNA‐based hybrid materials. These strategies expand the chemical scope of DNA nanotechnology and facilitate future applications in drug delivery and advanced materials science.

## Graphical abstract

This work introduces additional functionality into silica‐coated DNA origami nanostructures using non‐standard silica precursors. A fluorescent precursor enables enhanced intracellular tracking, while a disulfide‐containing reagent yields redox‐responsive, degradable silica coatings. These traceable and stimuli‐responsive hybrid nanostructures expand the application potential of DNA‐based silica materials.

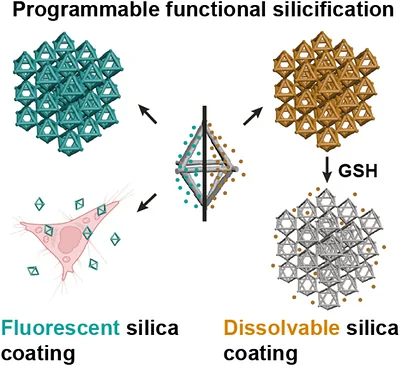

## 1 Introduction

Since its invention in 2006, the DNA origami technique [1] has become a versatile platform for a wide range of applications. It offers unprecedented shape programmability and a unique addressability for nanometer‐precise modifications with guest molecules such as fluorophores, metal nanoparticles or proteins. Among other potential fields of use, DNA origami nanostructures have been envisioned as templates for the growth of complex hybrid materials in material science or in biomedical applications [2, 3, 4, 5, 6]. Studies have also explored the distribution of DNA origami in cells (in vitro) [7] or living organisms (in vivo) [8]. However, there are several challenges associated with the biological applications of DNA origami for cargo transport or drug delivery. Apart from considerations such as efficient targeting or controlled release mechanisms, a major obstacle is the lack of stability of DNA origami in biologically relevant conditions where DNases and low salt condition lead to destabilization and degradation.

The stability of DNA origami against detrimental environmental influences can be improved by several techniques, including UV point‐welding, enzymatic ligation, or protective coatings such as virus capsid proteins, oligolysine‐PEG, or silica [9, 10, 11, 12, 13, 14]. Among those, the silicification of DNA origami (in solution or on surfaces, with coating thicknesses from sub‐nanometer to several nanometers) has become an established and efficient method to significantly increase DNA origami's chemical and biological stability against a variety of environmental cues [15, 16, 17, 18, 19, 20]. Importantly, silicification also preserves the designed shapes and precise addressability, since single‐stranded DNA (ssDNA) retains its hybridization ability even after silicification [21]. While the shape and mechanical properties of the nanostructures affect, for example, their cellular uptake behavior [22, 23, 24] as well as their ability to transport cargo, retaining addressability is of great importance for, for example, stimuli‐responsive structures. Additionally, amorphous silica is generally considered to be biocompatible [25], making silicified nanostructures ideal candidates for biological applications.

However, thus far, the silica coating itself has only served a passive, protective role. Here, we introduce a precursor‐engineered silicification approach that enables the programmable incorporation of functionality into silica coatings on DNA origami. Rather than modifying DNA prior to coating or post‐functionalizing silica, we embed functionality directly during shell formation by selecting and designing appropriate silane precursors. We demonstrate this concept using two complementary model systems: (i) a custom‐made fluorescein‐bearing silane for traceable silica coatings and a disulfide‐containing silane for redox‐responsive degradation. Using agarose gel electrophoresis (AGE) and confocal microscopy, we show that both single DNA origami as well as large DNA origami crystals can be fluorescently‐silicified. We also demonstrate that such fluorescently silicified structures can be tracked and remain stable inside cells for at least 24 h. On the other hand, we show that both single DNA origami structures as well as large DNA origami crystals endowed with a redox responsive silica coating are highly stable under otherwise detrimental conditions, but can be controllably degraded in the presence of a reducing agent such as glutathione (GSH) (Figure 1). When combined with the wide range of modifications possible on the DNA origami itself, such additional modifications offer a new and improved tool‐kit for silicified DNA origami nanostructures for various purposes, including future biomedical applications.

**FIGURE 1:**
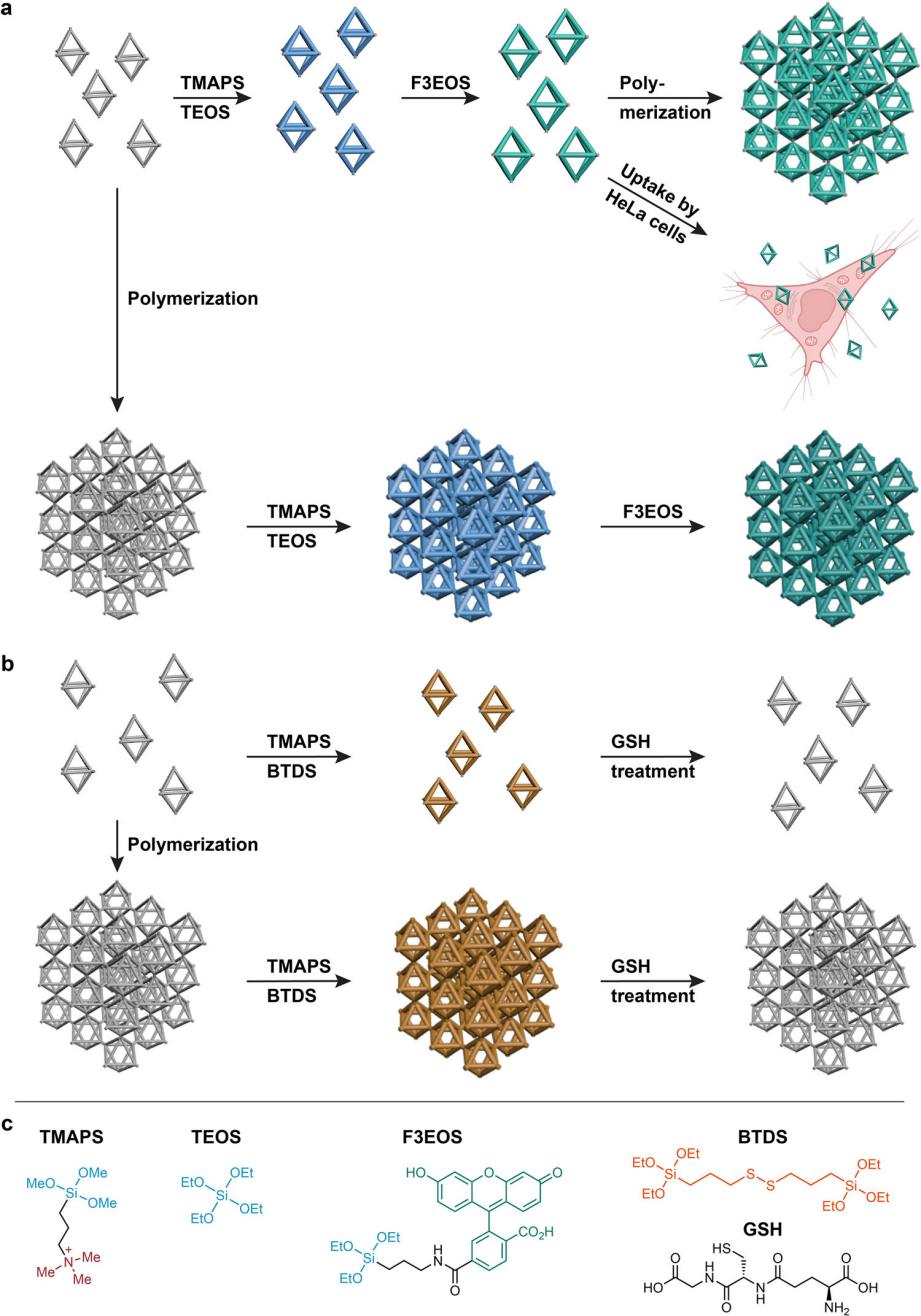
Schematic representation of the experimental workflow in this study. (a) Experiments with a fluorescent silane. (b) Experiments with a silica precursor containing a disulfide bridge. (c) Principal reagents used in this study.

## 2 Results and Discussion

### 2.1 Fluorescent Silica Coatings

Due to its high relevance in biological applications, our first goal was to introduce fluorescence to the silica coating. Generally, approaches to create fluorescent silica nanoparticles rely either on the physical entrapment of dyes in the pores or channels of a silica matrix [26], or on the covalent attachment of fluorescent dyes to silica precursors. Here we adapted the former approach to introduce fluorescence into the silica coating of DNA origami, which has not been shown to date. For this, we synthesized a fluorescein‐labelled silane precursor ‘F3EOS’, which can be incorporated during the silicification reaction, resulting in a stable fluorescent silica coating (Figure 1a), allowing, for example, the monitoring of structural integrity of silicified DNA origami nanostructures within cells. Tracking structural stability of DNA origami inside cells is rather challenging. Simple approaches such as tracking fluorescently modified staples within the DNA origami, only provide information about the presence of these fluorophores and their distribution in the sample, but do not report on the integrity of the entire nanostructure [27, 28]. Indeed, as shown by Lacroix et al., a single intracellular fluorescent signal may well originate from dissociated fluorophores or partially degraded nanostructures [29]. Other recently developed techniques such as origamiFISH [7] are able to overcome these limitations to an extent, but are not easily applicable to coated DNA origami structures. Therefore, endowing the silica coating with fluorescence would offer both enhanced stability, as well as the opportunity to monitor the silicified origami's structural integrity within a cellular environment.

Generally, the silicification of DNA origami relies on two main silica precursors. Initially, positively charged trimethyl[3‐(trimethoxysilyl)propyl]ammonium chloride (TMAPS) attaches electrostatically to the negatively charged DNA phosphate backbone, while the second precursor (usually neutral tetraethylorthosilicate (TEOS)) then forms a covalent silica network around the DNA origami nanostructure [14], not only forming an outer layer but also penetrating the internal structure of the DNA origami [15]. In order to introduce fluorescence into the silica coating, we synthesized a novel fluorescein‐conjugated neutral silane monomer F3EOS by acylating (triethoxysilyl)propylamine with carboxyfluorescein (Figure 1). Since F3EOS is an amide, we expected it to be chemically stable during silicification. However, we anticipated that using large amounts of F3EOS might increase the porosity of the silica network since it has one fewer binding site than TEOS. We also hypothesized that it might additionally slow the silicification due to the larger size of its fluorescein sidechain. Therefore, we developed a ‘two‐step‐silicification’ approach (Figure 1a): In the first step, we used the standard precursors TMAPS and TEOS to create a thin, stable initial silica coating around the DNA origami, followed by purification to remove excess precursor molecules. Then as the second step, a longer silicification with only F3EOS was performed to create the fluorescent outer silica layer.

We chose DNA origami octahedra for all silicification experiments, as these are versatile and well‐studied structures that can also be polymerized into cubic crystals via sticky end hybridization, even after initial silicification of the monomers [21] (see Figure S2 for the design). To allow us to identify both the DNA origami and the silica coating by separate fluorescence signals, a set of Cy5‐labelled staples was incorporated into the structures.

We then used the two‐step silicification procedure (cf. Figure 2a) to create fluorescently silicified Cy5‐labelled octahedral origami with an ultrathin silica coating. Analyzing the products using agarose gel electrophoresis (AGE) encouragingly showed that fluorescent F3EOS was successfully incorporated into the silica coating, and no significant unspecific binding of the fluorescent silane or its potential degradation product fluorescein to either bare or pre‐silicified DNA origami could be observed (Figures 2b and S3). We next tested the stability of the resulting structures with respect to heat and low salt conditions using AGE and transmission electron microscopy (TEM; Figures 2c). As expected, bare octahedra lost their structural integrity under these conditions, indicated by the upward shift in the gel bands and the loss in signal of the internal Cy5 modification. The fluorescently silicified nanostructures, however, did not show significant signs of degradation, and continued to exhibit a strong signal from both the internal Cy5‐labelled staples and the fluorescein‐containing silica coating, demonstrating the highly increased stability conferred by the silicification with F3EOS (Figures 2b and S4).

**FIGURE 2:**
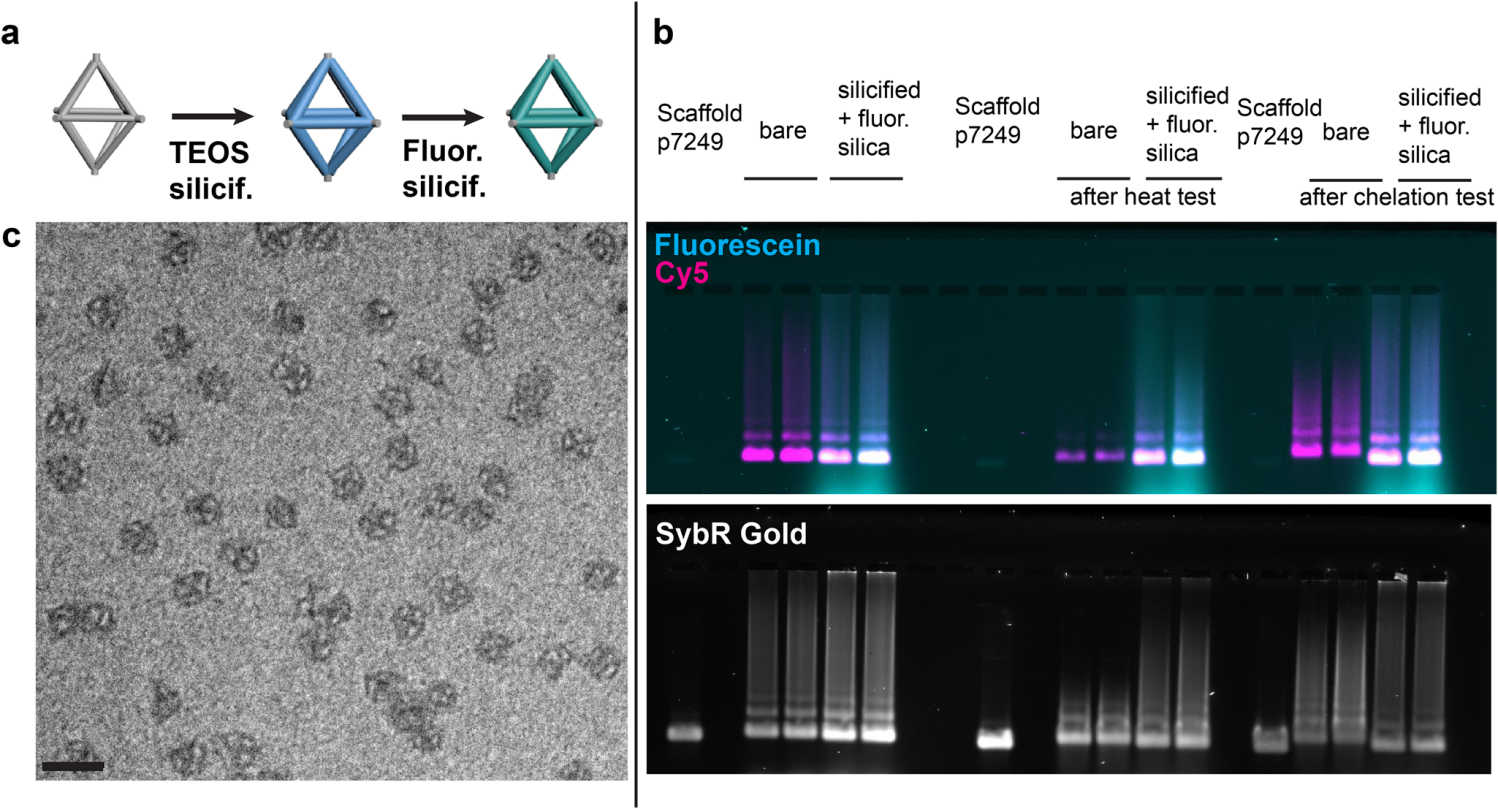
(a) Scheme depicting the experimental workflow for the fluorescent silicification of octahedral DNA origami monomers. (b) Agarose gel electrophoresis showing the successful integration of the fluorescent silane into the silica coating of the octahedral monomers and the enhanced stability of the silicified structures compared to bare octahedra. A quantitative analysis of the shown gel is depicted in Figure S4. (c) TEM image of fluorescently silicified octahedra after a heat test. Scale bar: 100 nm.

In order to further visualize the successful incorporation of F3EOS by confocal microscopy, we next used the silicified octahedral monomers to form micron‐sized cubic crystals (Figure 3a). For crystal formation, fluorescently silicified octahedra with two types of sticky ends (type A and B) were mixed and polymerized via a heat ramp. The resulting cubic crystals were then attached to a microscopy slide and imaged via confocal microscopy, showing strong fluorescent signal in both fluorescein and Cy5 channels (Figure 3b). Importantly, this also successfully showed that the fluorescent F3EOS remained incorporated even after undergoing a heat ramp for several days for crystal formation.

**FIGURE 3:**
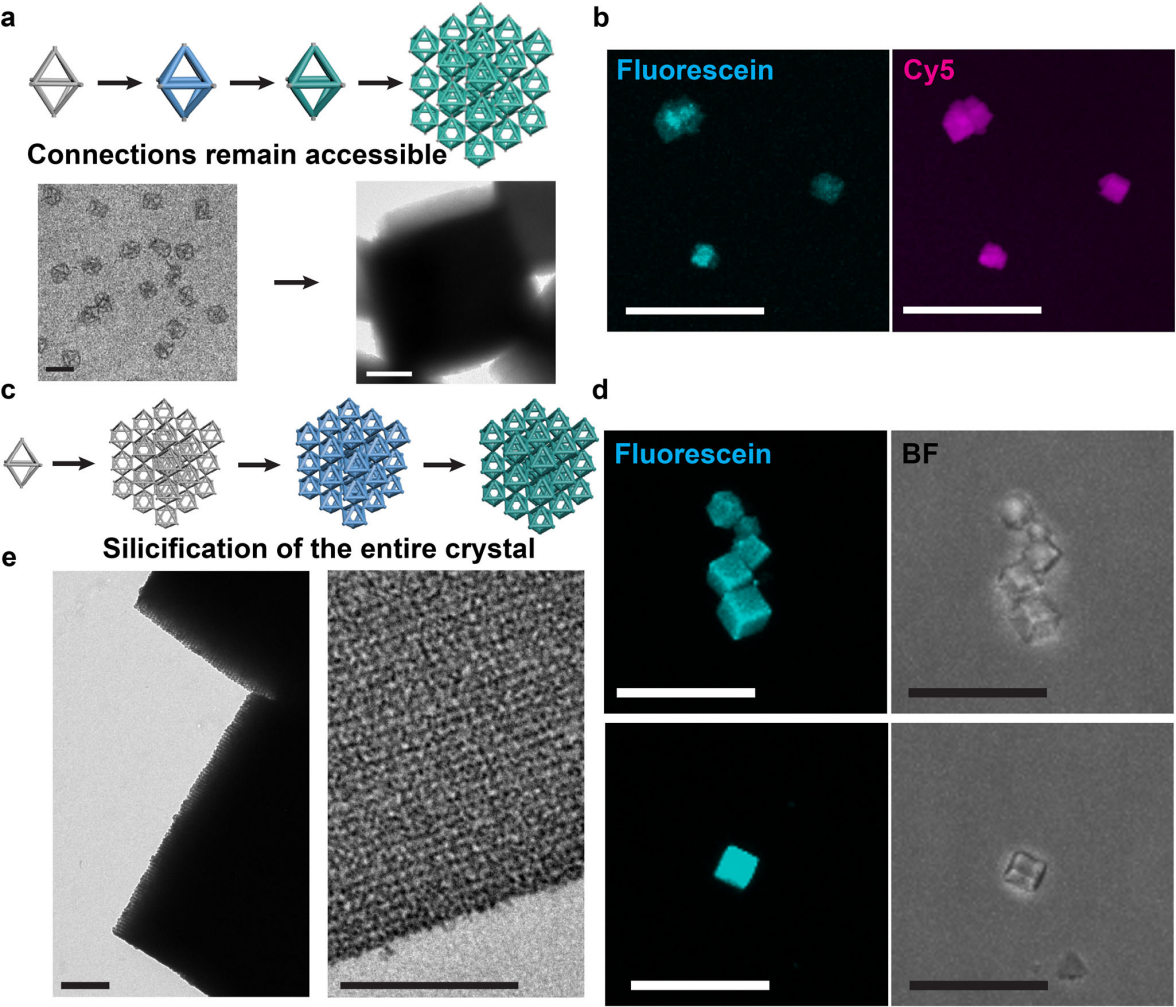
Fluorescent silicification of DNA origami crystal structures. (a) and (c) Schematic representation of the experimental workflows for the creation of silicified DNA origami crystals. (b) Confocal microscopy images of crystals obtained from the polymerization of fluorescently silicified octahedra. Scale bars: 20 µm. (d) 3D projections obtained from z‐stacks recorded using confocal microscopy. The imaged fluorescently silicified DNA origami crystals show a strong signal in the fluorescein channel, indicating the successful integration of a relatively large amount of fluorescent silica. Scale bars: 0.5 µm. (e) Left: TEM image showing a close‐up of the fluorescently silicified crystals. The strong contrast in the images is indicative of a thick silica coating. Right: Crystal slice showing the internal structure of the fluorescently silicified DNA origami crystals. The order within the crystals is maintained and the crystals appear to be homogeneously silicified. Scale bars: 20 µm.

This far we demonstrated that the fluorescent silane could be successfully incorporated into ultrathin silica coatings. To investigate the incorporation into much thicker coatings, we next applied the F3EOS silicification procedure to pre‐formed, bare DNA origami crystals (Figure 3c). We screened various reaction times for the crystal silicification (see Supplementary Note 1), finding that an addition of the F3EOS after 8‐10 h of ‘standard’ silicification with TMAPS and TEOS with a subsequent overnight incubation successfully led to a well‐distributed fluorescein signal throughout the entire crystal (not only on the edges), and a fully fluorescent cubic crystal structure, confirmed by Z‐scans through the entire crystals and 3D projections thereof (Figure 3d and S5, Supplementary Videos S1 and S2). TEM micrographs of entire crystals and thin sections showed a thick and uniform silica coating, yielding highly stable crystals that fully retained their lattice order upon exposure to cell culture medium, elevated temperatures and chelating conditions (Figure 3e and Figure S6).

After having established reliable conditions for the incorporation of fluorescence through F3EOS into silicified DNA origami, we next sought to test their ability to be used in biological applications. It was previously observed for silica nanoparticles that certain components of cell culture media can etch the silica layers within hours, causing dents in the surface and (partially) exposing core materials. This erosion was mostly attributed to the abundance of amino groups in the cell culture medium. However, it was also found that a higher level of polymerization within the silica network enhances the stability [30, 31]. In light of these observations, it is essential to investigate the stability of the silica coating itself in cellular environments. We therefore exposed bare, standard and fluorescently silicified origami to biologically relevant conditions and compared their stability. To enable direct comparison, all DNA origami structures without the fluorescent silane were equipped with both Cy5‐ and fluorescein (FITC) ‐modified staples, whereas the fluorescently silicified samples were equipped with Cy5‐carrying staples only. The incorporation of two different internal fluorescent modifications into the (bare) DNA origami samples allows for monitoring of the colocalization of these two signals, which can also serve as a tool for intracellular localization and allow for a qualitative assessment of structural integrity. Structures (bare, silicified and fluorescently silicified) were thus incubated in FluoroBrite DMEM cell culture medium with a final concentration of 10% fetal bovine serum (FBS) for up to 24 h and their stability analyzed by AGE (Figure S8). As expected, bare DNA origami exhibited clear signs of disassembly already after 2 h, however, both standard and fluorescently silicified samples displayed good stability even after 24 h. N.B.: the reaction time with F3EOS had to be increased compared to a standard silicification, as we found that even though structures remained stable, a strong release of the fluorescein signal could be observed after 24 h incubation in cell culture medium (Figure S9) after shorter silicification times, suggesting that our novel silica precursor requires significantly more time for the build‐up of a reasonably stable silica layer compared to TEOS. This could on the one hand be attributed to the reduced number of reactive groups (three instead of four), which can participate in the formation of siloxane bridges. And on the other hand, to the bulky fluorescein modification that could lead to steric hindrance and a reduced diffusion speed, resulting in a thinner, more porous fluorescent layer. It should be noted that even with the longer silicification time a slight reduction in the fluorescein signal could still be observed, indicating that some etching may take place, however, not negatively affecting origami stability.

After having successfully established the stability of our fluorescently silicified structures in cell culture medium, we next sought to investigate their behavior *in cellulo*. For this, bare and (fluorescently) silicified structures were incubated with HeLa cells and their uptake and intracellular stability were observed by confocal microscopy (see Figure 4 and Figure S10). To support the visual assessment, a CellProfiler pipeline was developed to automatically identify Cy5‐ and fluorescein‐foci and measure their intensity in both channels. Both intensities of each Cy5‐focus at the respective time point are plotted below each image. The coherent data for fluorescein‐foci is shown in Figure S11.

**FIGURE 4:**
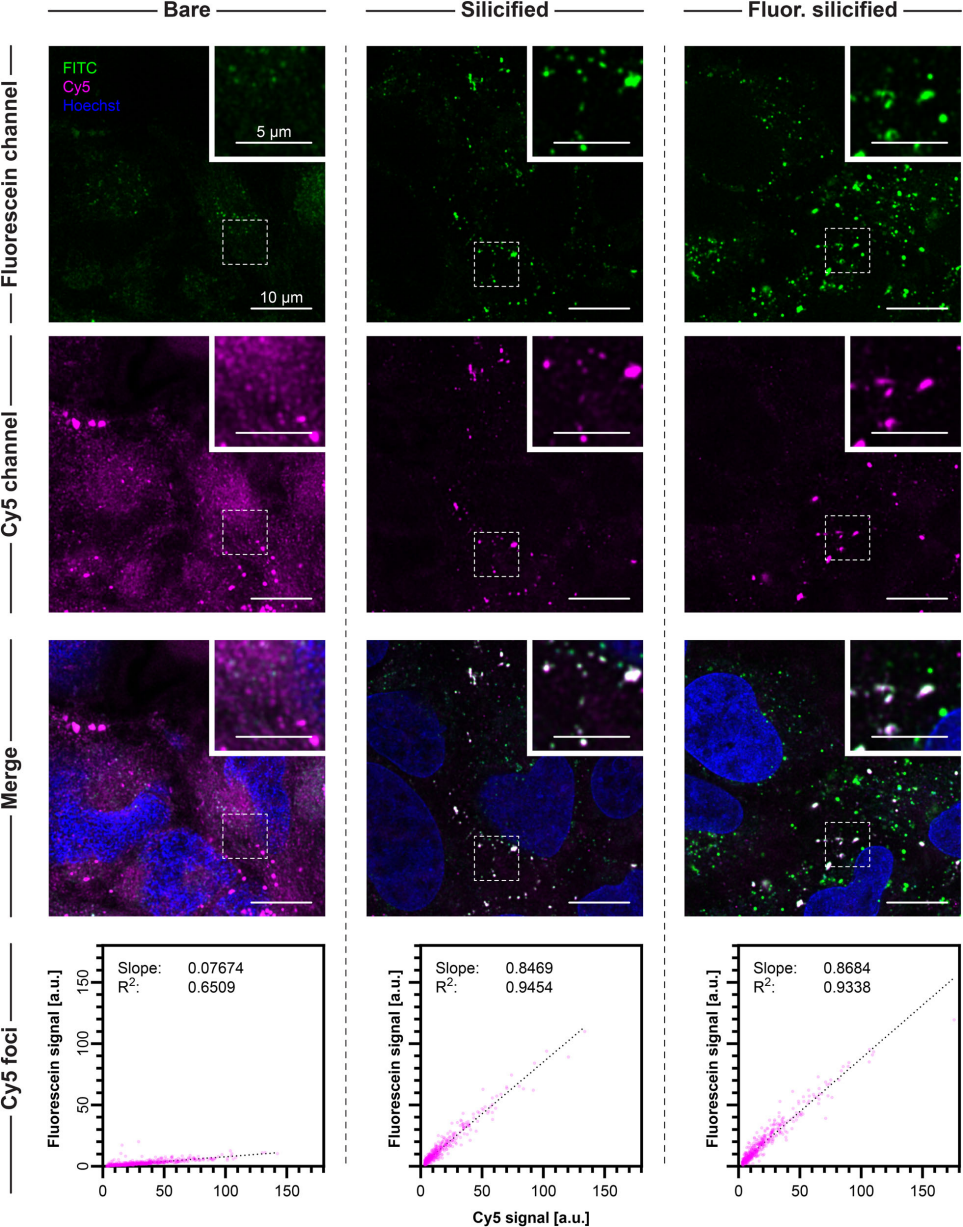
Cellular uptake of bare, silicified, and fluorescently silicified DNA origami nanostructures. Representative images are shown after 6 h (bare octahedra) or 24 h (silicified octahedra) of incubation with 5 nM DNA origami. For each detected Cy5‐focus at the respective time point, the measured Cy5 and fluorescein intensities are plotted below. Already after 6 h, the bare DNA origami nanostructures (first column) were clearly degraded. Both silicified and fluorescently silicified samples (second and third column) exhibited significant colocalization between the fluorescein and the Cy5 signal, indicating the stability of the structures even after 24 h. Scale bars: 10 µm, insets: 5 µm.

As can be seen from the confocal images in Figure 4, for bare structures no significant colocalization of the fluorescein and Cy5 signals (corresponding to the two modified, incorporated staples) could be observed after 6 h, indicating degradation of the structures as expected. Contrary to that, the silicified origami exhibited significant overlap of the two fluorescent signals even after 24 h, strongly suggesting that silicified structures remained largely intact. Encouragingly, also the fluorescently silicified samples exhibited a significant colocalization of the Cy5 and the fluorescein signal inside the cells even after 24 h of incubation. However, it has to be noted that while mostly all Cy5 signal colocalized with the fluorescein signal, some of the fluorescein signal did not overlap with any Cy5 signal, as indicated by two distinct populations in the Cy5‐fluorescein scatter plot. One population resembles strongly the silicified control, while the other one shows limited to no correlation between the measured fluorescein and Cy5 signals. This population was mostly found in a perinuclear localization, suggesting lysosomal accumulation. To investigate if the signal could arise from insufficient purification from the F3EOS precursor, we conducted control experiments where HeLa cells were incubated with F3EOS either directly from the stock solution or after a 4 d incubation with TMAPS and TEOS. As can be seen from the confocal images in Figure S12, no significant intracellular fluorescent signal could be observed after incubation with the F3EOS only. However, the control sample from a ‘mock silicification’ (TMAPS + TEOS + F3EOS, without DNA origami) yielded several bright fluorescent spots in the fluorescein channel, again mostly found in a perinuclear localization. We therefore hypothesize that the fluorescein signal originates mostly from small silica aggregates, which could not be fully removed during the purification steps. However, it cannot be fully excluded that some signal may also arise from a partially etched outer fluorescent silica layer from the DNA origami, in agreement with the AGE results shown in Figure S8. However, as the Cy5 signal showed excellent colocalization with the fluorescein signal, we infer that the structures remained intact, retaining their fluorescent coating even after 24 h. Importantly, we also found that all structures displayed excellent biocompatibility (see Figure S13).

These experimental results therefore establish the fluorescent silica precursor F3EOS as an effective tool for the investigation of the intracellular distribution and stability of silicified DNA origami nanostructures.

### 2.2 Degradable Silica Coating

In our second customization approach, we introduced the disulfide‐containing silane precursor bis[3‐(triethoxysilyl)propyl]disulfide (BTDS).It incorporates during silicification to give a DNA origami whose silica shell can be selectively disrupted by a reducing agent such as glutathione (GSH) (Figure 1b), making it a highly attractive approach for drug release applications, or for controllably‐degradable nanostructures that may alleviate concerns about organ toxicity due to nanoparticle accumulation [32, 33, 34]. For inorganic nanoparticles, different types of stimuli‐responsive shells have long been conceived and demonstrated, for example, nitrobenzyl‐containing coatings inferring responsiveness to UV light [35]. Among different responsive coatings, the integration of disulfide bridges is particularly attractive with regard to intracellular applications as they can easily be reduced in the presence of a reducing agent like GSH, which is present in cells in millimolar concentrations as a crucial antioxidant. Particularly cancer cells have been reported to exhibit elevated GSH levels and redox responsive coatings have been demonstrated for pure inorganic nanoparticles, small DNA tetrahedra as well as oligolysine‐PEG‐coated DNA origami [36, 37, 38, 39, 40, 41, 42]. However, so far, such redox responsive functionalization of the silica coating for DNA origami nanostructures has not yet been demonstrated; and stably incorporating the function‐carrying silica reagents into coatings with thicknesses ranging from below one to several nanometers without negatively affecting the protective effect of the coating had remained an unsolved challenge. To address this challenge, we employed BTDS, a silica precursor, that resembles a dimer of TEOS molecules, but contains a disulfide bridge (see Figure 1c). Upon reduction of the disulfide bridge, cracks in the silica coating are formed, making the underlying nanostructures again susceptible to environmental conditions. In order to create such a dissolvable silica coating, TEOS was fully replaced by BTDS during the silicification reaction. Interestingly, preliminary tests revealed that the standard silicification time of several hours was insufficient to create a protective coating (see Figure S14a,b). We hypothesize that the presence of the disulfide bond and the larger size of the molecule resulted in a slower reaction rate, therefore requiring the longer reaction time, similar to the F3EOS precursor. Therefore, the incubation time with both TMAPS and BTDS was extended to 4 d in order to achieve a stable coating. In a next step, we tested the degradability of the silica coating. For this, a sample containing BTDS‐silicified origami was incubated with a freshly prepared solution of GSH for 3 h. After a purification step to remove the GSH, the sample was subjected to both a chelation and a DNase I degradation test and was subsequently analyzed by AGE and TEM. As can be seen from Figure 5 and S15, BTDS‐silicified structures that were not pre‐treated with GSH successfully withstood the detrimental conditions and maintained their structural integrity both after chelation and DNase tests. However, if treated with GSH, structures were clearly degraded when subjected to chelating conditions or DNases, indicating successful dissolution of the silica coating and subsequent degradation of the DNA origami. Interestingly, even though GSH did not affect the structural integrity of the bare DNA origami, the nuclease digestion of GSH‐treated DNA origami was accelerated compared to untreated bare DNA origami (Figure 5d and Figure S16).

**FIGURE 5:**
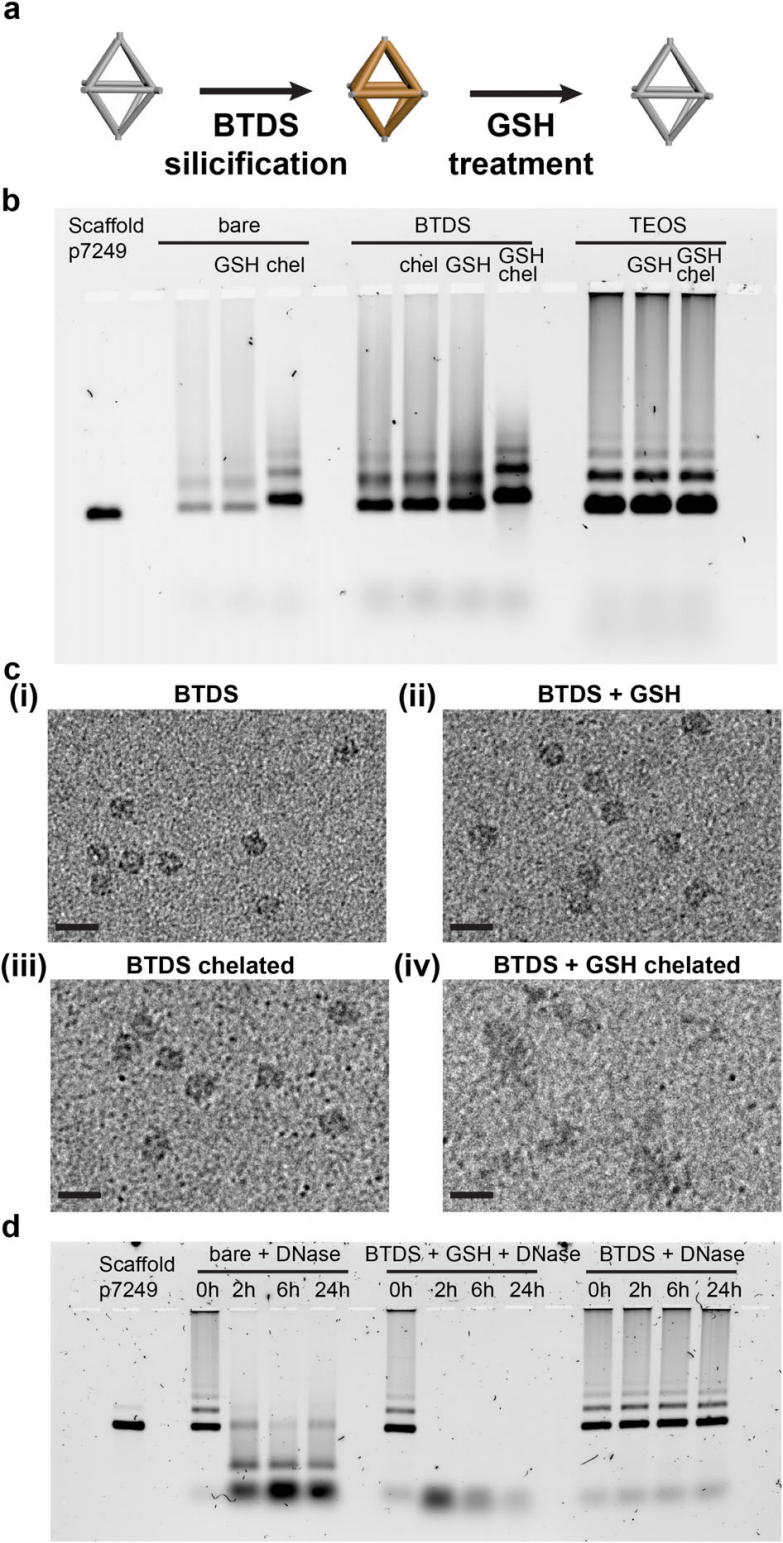
BTDS silicification of octahedral DNA origami monomers. (a) Scheme representing the BTDS silicification of octahedral monomers, followed by the incubation in GSH, leading to the dissolution of the silica coating. (b) Agarose gel showing the successful silicification of DNA origami octahedra with BTDS instead of TEOS. While the treatment with 10 mM GSH for 3 h does not seem to have a noticeable effect on samples with a standard silicification, the BTDS‐silicified and GSH‐treated sample clearly seems to be degraded during a chelation test, as can be seen from the upward shift of the main band. A similar behavior can be seen for the bare sample. (c) TEM images for (i) BTDS‐silicified monomers directly after purification, (ii) BTDS‐silicified monomers after treatment with GSH for 3 h, (iii) BTDS‐silicified octahedra after incubation in 1× TAE buffer for 3 h, and (iv) BTDS‐silicified and GSH‐treated octahedra which are disassembled. Scale bars: 100 nm. (d) DNase test for different DNA origami samples. While the bare DNA origami octahedra (left) are rapidly degraded by the DNases, the BTDS‐silicified monomers (right) can withstand even 24 h of incubation with the DNases. Interestingly, the BTDS‐silicified samples after GSH‐treatment exhibit an even faster and complete digestion already within the first 2 h of incubation with the DNase. A quantitative analysis of the shown gel can be found in Figure S15.

Taken together, our results show that the silicification with BTDS can indeed be used to create a silica coating that effectively shields the DNA origami nanostructures from detrimental conditions such as DNases and low salt environments, but that can also—unlike standard silica coatings—be dissolved in the presence of a reducing agent, creating hybrid DNA origami nanostructures with a stimuli‐responsive silica coating for the first time.

In order to test if the properties of the BTDS silicification also hold for large DNA origami superstructures with thicker silica coatings, we again formed cubic crystals, followed by BTDS‐silicification of the entire crystals. Crystals were then again incubated with GSH for 3 h followed by several washing steps, and subsequently subjected to a chelation test. As can be seen from the TEM images in Figure 6, the BTDS‐silicified crystals exhibit an increased contrast compared to bare crystals, indicating the successful formation of a distinct silica coating. As expected, BTDS‐silicified crystals that were not exposed to GSH were unaffected by the chelation test, successfully demonstrating that the BTDS‐silica coating is inferring sufficient stability. Encouragingly, after GSH‐treatment, TEM images of crystals showed a distinctly weaker contrast, similar to bare crystals, hinting at the dissolution of the silica coating. This was then further confirmed by a chelation test performed after GSH‐treatment, displaying the complete disassembly of the crystals in a low‐salt environment. We further compared results from Energy Dispersive X‐ray (EDX) analysis (Figure S17) acquired on a scanning TEM for BTDS‐silicified crystals pre‐ and post‐incubation with GSH. The measurements show a decrease in the amount of Silicon (normalized to the amount of Phosphorus) by 37% after GSH‐treatment, strongly suggesting partial degradation of the silica coating in response to the disruption of the disulfide bonds in the BTDS molecules, as desired. Together these results confirm that BTDS can be successfully used instead of TEOS for the silicification of both individual DNA origami as well as large superstructures, providing a protective coating against e.g. low salt environments, but allowing for the controlled dissolution of the silica network in a reducing environment.

**FIGURE 6:**
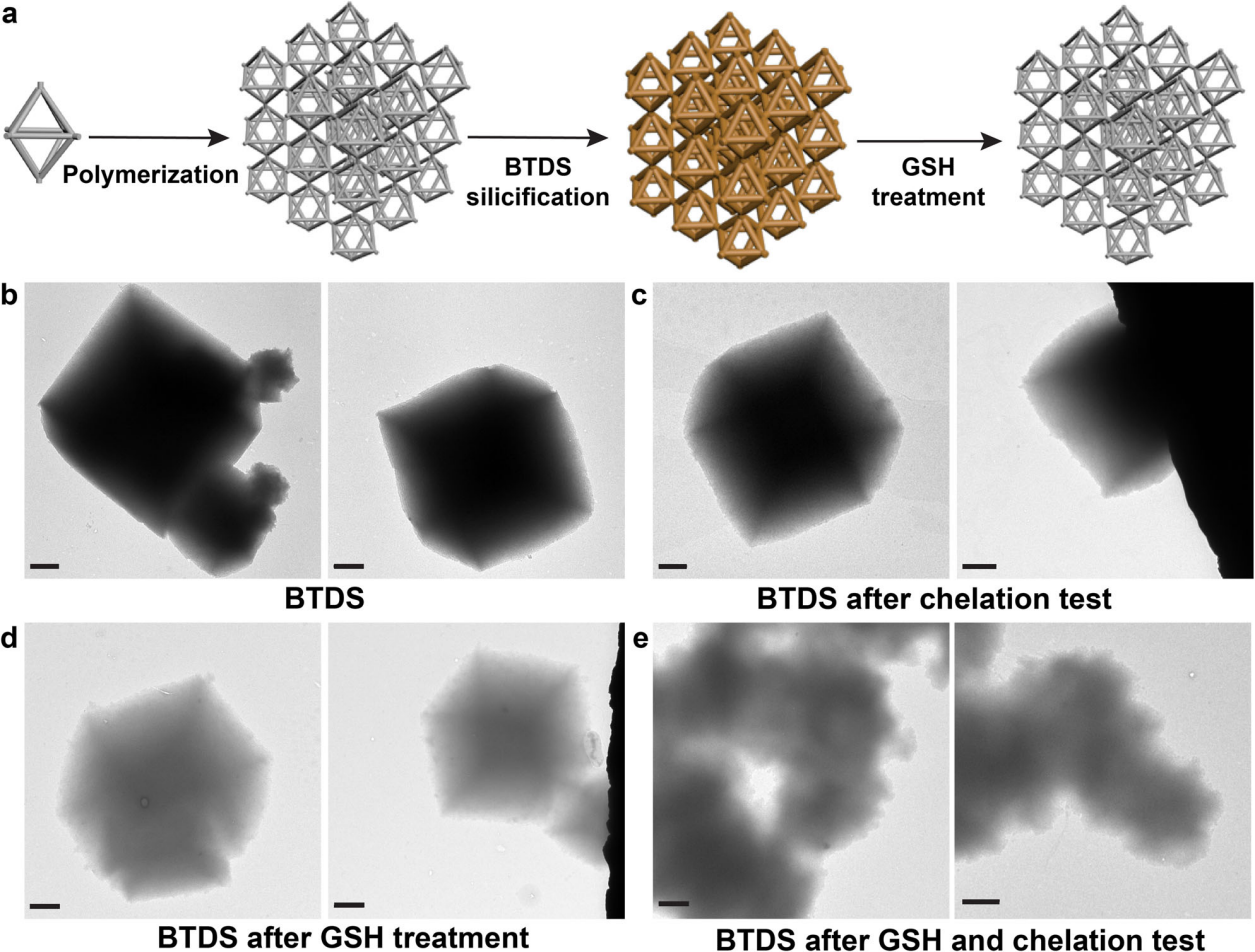
TEM images of BTDS‐silicified crystals. (a) Scheme representing the polymerization of bare octahedral monomers, the subsequent silicification with BTDS and the treatment of the silicified crystals, effectively disrupting the silica coating. (b) Crystals directly after silicification with BTDS and removal of excess reagents through washing. (c) BTDS‐silicified crystals after incubation in a 1× TAE buffer as a chelation test. (d) BTDS‐silicified crystals after treatment with 10 mM GSH for 3 h. (e) BTDS‐silicified crystals after treatment with GSH and subsequent incubation in 1× TAE. While the BTDS‐silicified crystals can withstand the chelating conditions, the GSH‐treated crystals disassemble during the chelation test, confirming the successful disruption of the BTDS‐silica coating by the reducing agent. Scale bars: 1 µm.

Importantly our findings also shed light on the behavior of non‐standard reagents during silica network formation. In the case of the dissolvable silica, BTDS resembles structurally a TEOS dimer with a connecting disulfide bridge, making the precursor substantially larger in size compared to TEOS. Similar to F3EOS with the comparably bulky fluorescein moiety, this appears to result in a slower diffusion speed and steric effects negatively affecting the speed of silica growth. Correspondingly, we found that significantly longer incubation times and higher molar excesses of BTDS were required. The differences in reactivity are also an important consideration when looking at mixtures of different precursors. When employing both TEOS and BTDS in a 1:5 molar ratio for crystal silicification we found that the coating was not dissolvable, suggesting that the majority of the coating did not contain BTDS (Figure S14c,d).

Thus, we conclude that for the introduction of any silane reagent different from TEOS in a DNA origami silicification, the chemical structure and its potential effects on the reactivity of the reagent and thus on silica network formation have to be taken into account and carefully tuned. Having laid the foundations for such work, we anticipate that this study provides important insights for the future use of other silane precursors.

## 3 Conclusion

In this work, we established customizable silicification as a versatile strategy to transform DNA origami nanostructures into multifunctional hybrid materials that can aim at impactful applications. By integrating non‐standard silane precursors directly into the sol‐gel process, we demonstrated that silica coatings can be endowed with additional, customizable functionality, rather than serving solely as passive protective shells.

Using the novel fluorescein‐labelled silane F3EOS, we generated fluorescent silica coatings on both individual DNA origami and large cubic crystals, enabling direct visualization of the inorganic shell and, critically, providing a new means to assess nanostructure integrity in complex biological environments. This approach overcomes longstanding limitations associated with tracking DNA origami *in cellulo* and reveals that functionalized silica coatings can retain their protective property while adding diagnostic capability. In parallel, incorporation of the disulfide‐bridged silane BTDS yielded a redox‐responsive silica network that remains stable under extracellular conditions yet can be selectively disrupted in reducing environments.

Beyond introducing fluorescence and on‐demand degradability as proof‐of‐concept functionalities, our findings also point out design principles for programmable silicification on DNA origami which should be carefully observed for each new precursor: (i) The size and steric nature of the precursor may influence coating homogeneity and surface coverage and (ii) the precursor reactivity appears to strongly influences incorporation into the growing silica network. We anticipate that our results will serve as the basis for further studies on the proceeding/kinetics of silicification reactions and that they can be of great relevance for establishing practical rules for expanding the palette of silicified DNA origami hybrid nanostructures. Looking forward, the findings presented in this work provide a foundation for extending silicification to a broader range of functional silanes, enabling tailored material properties incorporating, for example, optical, chemical, or stimuli‐responsive motifs through molecular design. We anticipate that programmable silicification will enable the development of tailored inorganic coatings for applications in nanotechnology, sensing and biointerfaces. For translation toward in vivo applications, several important challenges remain. Future studies will need to establish how different silica chemistries influence biodistribution, protein corona formation, immune recognition, long‐term clearance and targeting performance in complex biological environments, while also developing predictive design rules for the incorporation of new functional silane precursors. Addressing these challenges will be crucial for the rational development of application‐specific silicified DNA origami nanostructures and will further expand the versatility of programmable silicification as a platform for functional hybrid materials.

## 4 Experimental Methods

### 4.1 Synthesis of Fluorescent Silica Precursor F3EOS

#### 4.1.1 Analytical Methods

High resolution mass spectrometry (**HRMS**) was conducted using a Thermo Finnigan LTQ FT Ultra Fourier Transform ion cyclotron resonance spectrometer from Thermo Fisher Scientific GmbH applying electron spray ionization (ESI) with a spray capillary voltage of 4 kV at temperature 250°C with a method dependent range from 50 u to 2,000 u.

Nuclear magnetic resonance (**NMR**) spectroscopy was performed using a Bruker Avance III HD Biospin (400/100 MHz, with BBFO cryoprobe) from Bruker Corp. at 400 MHz. NMR‐spectra were measured at 298 K, and were analyzed with the program MestreNova 14 developed by MestreLab Ltd. ^1^H‐NMR spectra chemical shifts (*δ*) in parts per million (ppm) relative to tetramethylsilane (*δ* = 0 ppm) are reported using the residual protic solvent (CHD_2_OD in CD_3_OD: *δ* = 3.31 ppm) as an internal reference. For ^13^C‐NMR spectra, chemical shifts in ppm relative to tetramethylsilane (*δ* = 0 ppm) are reported using the central resonance of the solvent signal (CD_3_OD: *δ* = 49.00 ppm) as an internal reference. For ^1^H‐NMR spectra in addition to the chemical shift the following data is reported in parenthesis: multiplicity, coupling constant(s) and number of hydrogen atoms. The abbreviations for multiplicities and related descriptors are s = singlet, d = doublet, t = triplet, q = quartet, or combinations thereof, m = multiplet and br = broad.

Analytical high performance liquid chromatography (**HPLC**) analysis was conducted using an Agilent 1200 SL system Agilent Technologies Corp., Santa Clara (USA) equipped with a DAD detector, a Hypersil Gold HPLC column from Thermo Fisher Scientific GmbH, Dreieich (Germany) and consecutive low‐resolution mass detection using a LC/MSD IQ mass spectrometer applying ESI from Agilent Technologies Corp., Santa Clara (USA). For both systems mixtures of water (analytical grade, 0.1% formic acid) and MeCN (analytical grade, 0.1% formic acid) were used as eluent systems.

**UV–vis** spectra were recorded on a Cary 60 UV–vis spectrophotometer from Agilent Technologies Inc., Santa Clara (USA) using 1 cm quartz or PMMA cuvettes. The scan rate was set to 600 nm/min and 2.5 nm slit width was used. Unless stated otherwise, the probes and fluorophores were dissolved in PBS (pH 7.4, 1% DMSO) at 10 µM concentration.

**Fluorescence spectroscopy** was performed on a Cary Eclipse Fluorescence Spectrometer from Agilent Technologies Inc., Santa Clara (USA) using quartz cuvettes. 480 nm light was used for excitation and spectra were recorded from 490−620 nm. The scan rate was set to 120 nm/min and 5 nm slit width was used. Unless stated otherwise, the probes and fluorophores were dissolved in PBS (pH 7.4, 1% DMSO) at 10 µM concentration.

All reactions were performed without precautions in regard to potential air‐ and moisture‐sensitivity and were stirred with Teflon‐coated magnetic stir bars. For solvent evaporation a Laborota 400 from Heidolph GmbH, Schwabach (Germany) equipped with a vacuum pump was used. Reactions were monitored by analytical HPLC.

All chemicals, which were obtained from BLDPharm, Sigma–Aldrich, TCI, Alfa Aesar, Acros, abcr, or carbolution, were used as received and without purification. Tetrahydrofuran (THF), dichloromethane (DCM) and dimethylformamide (DMF) were provided by Acros and were stored under argon atmosphere and dried over molecular sieves. TLC control, extractions and column chromatography were conducted using distilled, technical grade solvents.

#### 4.1.2 Synthesis Procedure

Two regioisomers were used in this paper, synthesized by two synthetic procedures: (1) Amide coupling in DMSO which can be directly used for silicification, and (2) Amide coupling in DMF followed by extraction to remove the *N*‐hydroxy succinimide side product. Generally, we recommend using Procedure 1 as it avoids silicification during the product isolation.

### 4.2 Synthesis Fluorescein‐5‐Silane (S01)


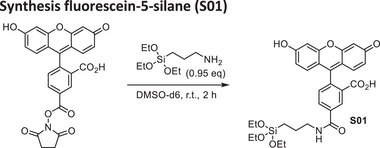


(3‐Aminopropyl)triethoxysilane (7.1 µL, 30 µmol, 1.0 equiv.) was diluted with anhydrous, perdeuterated dimethyl sulfoxide (DMSO‐d6, 93 µL) and added to 5‐carboxyfluorescein *N*‐succinimidyl ester (purchased from TCI, 15 mg, 32 µmol, 1.05 equiv.). The mixture was heated to 37°C in a heat bath for 5 min and then incubated at room temperature for 2 h (without stirring). After full conversion, DMSO‐d6 (500 µL) was added to obtain a 50 mM stock solution of **S01**.

**^1^H‐NMR** (400 MHz, DMSO‐d6): *δ* (ppm) = 8.83 (t, *J* = 5.6 Hz, 1H), 8.52 – 8.42 (m, 1H), 8.24 (dd, *J* = 8.1, 1.5 Hz, 1H), 7.37 (d, *J* = 8.1 Hz, 1H), 6.69 (d, *J* = 2.2 Hz, 2H), 6.59 (d, *J* = 8.7 Hz, 2H), 6.55 (dd, *J* = 8.7, 2.2 Hz, 2H), 3.76 (q, *J* = 7.0 Hz, 6H), 3.29 (q, *J* = 6.8 Hz, 2H), 1.61 (p, *J* = 7.9 Hz, 2H), 1.15 (t, *J* = 7.0 Hz, 9H), 0.65 – 0.58 (m, 2H); **^13^C‐NMR** (101 MHz, DMSO‐d6): *δ* (ppm) = 168.3, 164.5, 159.7, 154.6, 151.9, 136.4, 134.7, 129.2, 126.5, 124.3, 123.3, 112.7, 109.1, 102.3, 83.4, 57.8, 42.2, 22.6, 18.3, 7.5.

### 4.3 Synthesis of Fluorescein‐6‐Silane (S02)


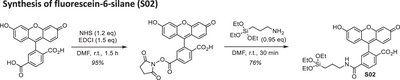


6‐carboxyfluorescein NHS‐ester was prepared following a previously described procedure [43]. 6‐Carboxyfluorescein (purchased from BLDPharm, 30 mg, 80 µmol, 1.0 equiv.) was dissolved in anhydrous *N*,*N*‐dimethyl formamide (2 mL). 1‐Ethyl‐3‐(3‐dimethylaminopropyl)carbodiimide hydrochloride (EDCI, 23 mg, 0.12 mmol, 1.5 equiv.) and *N*‐hydroxysuccinimide (11 mg, 96 µmol, 1.2 equiv.) were added and the reaction was stirred at room temperature for 1.5 h. The reaction mixture was diluted with ethyl acetate (20 mL) and washed with phosphate buffer (pH 6, 2× 20 mL). The organic layer was dried over sodium sulfate, the desiccant was filtered off and the volatiles were removed *in vacuo* to give 6‐carboxyfluorescein NHS‐ester (36 mg, 76 µmol, 95%) as an orange solid.

The obtained 6‐carboxyfluorescein NHS‐ester was dissolved in anhydrous *N*,*N*‐dimethyl formamide (2 mL). (3‐Aminopropyl)triethoxysilane (17 µL, 71 µmol, 0.95 equiv.) was added and the reaction mixture was stirred at room temperature for 30 min. The mixture was diluted with ethyl acetate (30 mL) and water (30 mL) and the layers were separated. The organic layer was dried over sodium sulfate, the desiccant was filtered off and the volatiles were removed *in vacuo* to give the title compound (**S02**, 30 mg, 52 µmol, 76%) as an orange solid.

Note: the reaction was monitored by HPLC analysis regarding consumption of the NHS‐ester. The product however decomposes during the HPLC run to partially reveal the de‐ethylated R‐Si(OH)_3_ decomposition product. This does not correspond to decomposition during the reaction as confirmed by NMR analysis of the product.

**^1^H‐NMR** (400 MHz, MeOD): *δ* (ppm) = 8.13 (dd, *J* = 8.0, 1.4 Hz, 1H), 8.09 – 8.06 (m, 1H), 7.60 (s, 1H), 6.69 (d, *J* = 2.3 Hz, 2H), 6.61 (d, *J* = 8.7 Hz, 2H), 6.54 (dd, *J* = 8.7, 2.4 Hz, 2H), 3.75 (q, *J* = 7.0 Hz, 6H), 3.31 – 3.24 (m, 2H), 1.69 – 1.58 (m, 2H), 1.15 (t, *J* = 7.0 Hz, 9H), 0.65 – 0.56 (m, 2H); **^13^C‐NMR** (101 MHz, MeOD): *δ* (ppm) = 170.6, 168.2, 161.4, 155.0, 154.1, 142.5, 130.3, 130.3, 130.3, 126.2, 123.9, 113.8, 110.9, 103.6, 85.7, 59.5, 43.7, 23.7, 18.6, 8.6; **HRMS** (ESI−): *m*/*z* calc. for C_30_H_32_NO_9_Si^−^ [M−H]^−^: 578.1852, found: 578.1851.

### 4.4 Folding and Purification of DNA Origami Octahedra

The DNA origami octahedra used here were designed using the TALOS [44] and CaDNAno [45] software (design schematics in Figure S2).

The folding of the octahedra was carried out using 20 nM of the scaffold p7249 and 100 nM of each staple strand in buffer containing 40 mM Tris, 20 mM acetic acid, 1 mM EDTA (pH 8.0) and 15 mM MgCl_2_. Two separate mixtures were prepared, containing the two different sets of end staples (type A or type B). The mixtures were heated to 95°C and held at this temperature for 1 min and then cooled down to 20°C over a period of 20 h. The fully assembled DNA origami nanostructures were purified from excess staple strands via ultrafiltration (Amicon centrifugal filter units, 0.5 mL, 100 kDa cut‐off). The folding mixtures were loaded into pre‐wetted filter units (type A and B separately, 250 µL per filter unit together with 250 µL of fresh buffer) and centrifuged at 2,000 rcf for 20 min. The centrifugal steps were repeated 5 times, whereby fresh buffer (1× TAE, 7.5 mM MgCl_2_, 400 µL) was added in every step. Finally, the DNA origami were eluted by placing the inverted filter unit into a new collection tube and spinning the tube for 8 min at 2,000 rcf.

The successful folding of the octahedral nanostructures was confirmed by TEM imaging.

### 4.5 Polymerization of Octahedral DNA Origami Monomers/DNA Origami Crystal Formation

Assembly of cubic crystals from octahedral DNA origami monomers was achieved by mixing the two different monomer types (A and B, at least 15 nM each, either both bare or both silicified) in a 1× TAE buffer containing 25 mM MgCl_2_. The reaction mixture (total volume 40 µl) was heated to 48°C for 1 h and then slowly and gradually cooled down to 20°C over a period of around 70 h (−1°C per 150 min).

### 4.6 Silicification of DNA Origami Octahedra (Monomers)

The silicification of octahedral DNA origami monomers was performed similarly to the procedure described previously [21]. 50 µL of purified DNA origami sample were prepared at a concentration of 50 nM in buffer containing 1× TAE, 7.5 mM MgCl_2_ in a round‐bottom Eppendorf tube. The tube was placed on a thermo shaker and the first silica precursor TMAPS (TCI, diluted 1:19 in methanol) was added to the solution in a sixfold molar excess to the number of nucleobases. After one minute of shaking at 400 rpm at 21°C, the second silica precursor TEOS (Sigma Aldrich, diluted 1:9 in methanol) was added to the sample in a 15‐fold molar excess to the number of nucleobases. The reaction mixture was left on the thermo shaker for 15 min at 400 rpm and was then transferred to a V‐bottom tube and put on a tube revolver rotator (Thermo Scientific) and rotated at 40 rpm at room temperature (21°C) for 6 h. Subsequently, the sample was purified from excess silica via buffer exchange in spin filtration columns (Zeba Spin Desalting Columns, 0.5 mL, 40 kDa cut‐off). After the storage solution was eluted from the spin columns (1,500 rcf, 1 to 2 min), they were washed three times with fresh buffer (1× TAE, 7.5 mM MgCl_2_). Then, the spin columns were placed in a fresh collection tube and the sample was added into the columns. After centrifugation at 1,500 rcf for 2 min, the eluted sample was collected.

For fluorescent silicification of the DNA origami octahedra, the silicified (and purified) sample (ca. 50 µL, 45 nM) was placed into a light‐protected V‐bottom tube and 7.5 µL of fluorescent silica precursor (10 mM) were added. After careful mixing of the solution, the tube was again placed on the tube revolver rotator and rotated at 40 rpm for the desired incubation time (3 d). Purification was carried out using up to two rounds of spin filtration, as described above.

For the BTDS‐silicification of the octahedral monomers, the BTDS was added undiluted to the solution on the thermo shaker in a 125‐fold molar excess to the number of nucleobases instead of the TEOS. Furthermore, the incubation time on the tube revolver rotator was extended to 4 d.

### 4.7 Silicification of DNA Origami Crystals Made From Octahedral Monomers

A sample with freshly polymerized DNA origami crystals (40 µl, 30 nM monomers) was put into a round‐bottom tube and the tube was placed on a thermo shaker at 4°C and shaken at 700 rpm. 0.75 µl of the first silica precursor TMAPS (diluted 1:9 in methanol) were added to the solution and the sample was left shaking for 30 min. Then, 0.75 µl of the second silica precursor TEOS were added to the sample and the shaking was continued for another 210 min. The temperature was increased to 10°C, while the shaking speed was kept at 700 rpm for another 2 h. After that, the temperature was further raised to 16°C and the shaking was continued for 2 h. Following that, the temperature was increased to room temperature (21°C) and the shaking speed was reduced to 300 rpm. The sample was left shaking overnight (16 h, total time 24 h).

For the purification of the silicified crystals, the sample was transferred to a V‐bottom tube and 300 µl of buffer solution were added. Then, the sample was left undisturbed, so that the silicified crystals could sediment to the bottom of the tube. In one washing step, the supernatant (ca. 300 µl) was carefully removed from the top of the solution, followed by a refilling of the buffer solution. For each sample, at least 3 washing steps were carried out.

For fluorescent silicification of the crystals, 1 µL to 7.5 µl of fluorescent silica precursor (10 mM) were added during the shaking (see Figure S6, optimal time point: 10 h after start of the silicification procedure). Purification was carried out by transferring the sample into a new V‐bottom tube and adding 300 µL to 500 µl of fresh buffer (1× TAE, 7.5 mM MgCl_2_). The sample was left undisturbed until the silicified crystals sedimented to the bottom of the tube. Then, the liquid above the crystals was very carefully removed and the tube was re‐filled with fresh buffer. This washing step was repeated at least twice. All samples containing Cy5 and/or fluorescent silica were kept in the dark/in light‐protected tubes.

For the fabrication of a dissolvable silica coating, the TEOS was replaced by 1.86 µl of undiluted BTDS that was added undiluted after 30 min of incubation on the thermo shaker.

### 4.8 Stability Tests

Heat testing of the silica coating (standard and fluorescent) was carried out by incubating the sample at 60°C for 30 min (monomers) or 60 min (crystals) in a thermocycler.

Chelation tests were performed by incubating the samples in MgCl_2_‐free 1× TAE buffer for at least 3 h. The buffer exchange was done using Zeba spin filtration columns (0.5 mL, 7 kDa cut‐off, 1,500 rcf).

### 4.9 Agarose Gel Electrophoresis

DNA origami samples (15 µl, 10 nM in 1× TAE buffer with 7.5 mM MgCl_2_) were mixed with loading buffer containing Orange G and Ficoll and loaded onto a 0.7% agarose gel (1× TAE, 11 mM MgCl_2_). The gel was run on ice for 120 min at 75 V (running buffer: 1× TAE with 11 mM MgCl_2_). Gel imaging was subsequently carried out using the Typhoon FLA‐9000 (GE Healthcare). For post‐staining, the gel was incubated in 100 mL MilliQ water with 0.01% SYBR Gold for 20 min and then thoroughly washed in MilliQ water, followed by another round of imaging.

Quantitative analysis of AGE was done by plotting the intensity profile in Fiji 2.16 [46], and further processing the received data in Excel and GraphPad Prism 11.0.

### 4.10 Transmission Electron Microscopy

Monomers: A DNA origami sample (10 µl, 10 nM) was applied to a carbon‐coated copper grid (300‐mesh, Plano GmbH) that had been plasma‐cleaned for 30 s. Silicified DNA origami samples were incubated on the TEM grids for 6 to 8 min. In the next step, the remaining solution was carefully removed from the grid using filter paper and the grid was washed with MilliQ water (5 µl each) twice and dried in air before imaging.

Crystals: The DNA origami crystals were collected from the bottom of a tube and the sample (10 µL) was then incubated on a plasma‐cleaned (30 s) TEM grid for 45 to 55 min. Subsequently, the remaining liquid was carefully removed with filter paper and the grid was washed twice with MilliQ water (5 µl each) and then air‐dried before imaging.

Preparation of crystal slices: The silicified DNA origami crystals were collected from the bottom of a tube (10 µl) and placed into a fresh V‐bottom tube. A solution containing 4% agarose was prepared and kept on a heat plate for a few minutes to avoid solidification and remove as much air as possible from the solution. Subsequently, approximately 100 µl of the agarose solution were carefully poured into the tube that contained the sample in order to encapsulate the DNA origami crystals in the agarose. Then, the sample was left at room temperature for full solidification. In the next step, small cubes (2 mm × 2 mm × 2 mm) containing the crystals were cut out. These cubes were then successively immersed in increasing amounts of ethanol (30%, 50%, 70%, 96%, 2× 100%) for 20 min per step, followed by the embedding in Spurr's low viscosity embedding medium according to the manual. After the embedding, the crystals were localized in the resin block and a small pyramid (500 µm × 500 µm) was trimmed using a razorblade. Then, slices with a thickness of about 50 to 60 nm were cut from these pyramids with a Diatome Diamond Knife (Ultra 45°) using an Ultramicrotome (Leica UC6). The resulting slices were collected on Formvar coated copper slot grids (Plano GmbH).

All images were recorded on a Jeol‐JEM‐1230 TEM operating at an accelerating voltage of 80 kV with a Gatan Orius SC1000 digital camera (software: Gatan DigitalMicrograph). The images were subsequently analyzed using the ImageJ software [47].

Energy Dispersive X‐ray (EDX) spectroscopy was conducted on a ThermoFisher Talos F200X G2 TEM in scanning mode with an acceleration voltage of 200 kV. The Images were analyzed using the proprietary software Velox from ThemoFisher.

### 4.11 Confocal Microscopy

The samples for confocal microscopy were prepared in a six‐channel ibidi slide. First, 100 µl of BSA‐biotin (1 mg/mL, dissolved in buffer containing 10 mM Tris, 100 mM NaCl, pH 8) were incubated in each channel for 10 min, followed by washing with 450 µl of buffer. After that, 100 µl of neutravidin (0.5 mg/mL in buffer containing 10 mM Tris, 100 mM NaCl, pH 8) were added to the channels for 10 min. Following this, the channels were washed with 300 µl of buffer containing 10 mM Tris and 100 mM NaCl and then with 450 µl of buffer containing 1× TAE and 7.5 mM MgCl_2_. In the next step, the biotinylated DNA origami samples were incubated in the channels for 30 min. Subsequently, the channels were flushed with 450 µl buffer (1× TAE, 7.5 mM MgCl_2_) and the slide was kept in the dark until the imaging.

The imaging was carried out on a Leica Stellaris 5 confocal microscope with a 63× (NA 1.4) oil‐immersion objective. The obtained images were analyzed with Fiji [46].

### 4.12 Cell Culture

HeLa cells (ACC 57, acquired from Leibniz Institute DSMZ) were cultured in DMEM (with high glucose, pyruvate, GlutaMax, added Penicillin and Streptomycin and 10% FBS) at 37°C and 5% CO_2_ and reseeded every two to three days with a ratio of 2×10^5^ to 5×10^5^. Cell detachment was conducted using Trypsin/EDTA. During each split, cell count and viability were determined using Trypan Blue and an automatic cell counter. Not more than 15 passages were conducted after receiving the cell line.

### 4.13 Uptake Experiments

Two days prior to t_0_, 5,000 HeLa cells were seeded in 50 µL DMEM (with high glucose, pyruvate, GlutaMax, added Penicillin and Streptomycin and 10% FBS) in an ibidi 15 well µ‐slide and incubated at 37°C and 5% CO_2_. One day prior to t_0_ the medium was exchanged by FluoroBrite DMEM (supplemented as DMEM). Subsequently, a given volume of medium was removed and exchanged by DNA origami in DPBS with the Mg^2+^ concentration adjusted to 2.5 mM for the 24 h incubated sample. Shorter incubation times were realized by adding the origami suspension at a later time point. The origami concentration in buffer was adjusted to be 30 nM, the exchanged volume was chosen to result in 5 nM final origami concentration in medium. At t_0_ the cells were washed with cold DPBS (with Ca^2^^+^ and Mg^2+^) and subsequently incubated in Image‐iT 4% Paraformaldehyde solution for 15 min at room temperature. Afterwards, the wells were washed with DPBS with 500 ng/mL Hoechst 33342 and again incubated for 5 min at room temperature. After a final wash with DPBS, the cells were imaged using a Leica Stellaris 5 confocal microscope with a 63× (NA 1.4) oil‐immersion lens.

Images were deconvoluted using SVI Huygens Essential 25.04 (https://svi.nl) and analyzed in Fiji 2.16 [46] and CellProfiler 4 [48]. Fiji was used to perform a maximum intensity projection (along z‐axis), split channels and to convert the images from 32bit float to 16bit integer data. CellProfiler was used to detect Cy5‐ and fluorescein‐foci using a size‐gated global intensity threshold after top‐hat transformation. Detected foci were filtered to exclude DNA origami aggregates or off‐target clusters from the final analysis. Affiliation to a cell was estimated by the distance to detected nuclei and the intensity in both Cy5 and fluorescein channel measured for each focus. For the used modules and parameters see Figure S18.

### 4.14 Cell Viability

Cell viability was determined using the cell counting kit 8 (CCK8) acquired from abcam (ab228554). The cytotoxicity of different silicified origami samples was conducted according to the provided protocol in a 96‐well plate. 10^4^ cells in DMEM (supplemented as before) were seeded per well and after 1 d, the medium was changed to 80 µL fresh DMEM and 16 µL origami samples in DPBS with 2.5 mM Mg^2+^ (5 nM final concentration in well) were added and co‐incubated for 24 h. Subsequently, 10 µL CCK8‐reactant were added and the absorbance at 450 nm was measured after 4 h.

The data was tested for outliers using Grubbs’ test (*α* = 0.05) in GraphPad Prism 11.0. Subsequently, the blank was subtracted from each sample followed by normalization to a DMEM only control.

## Conflicts of Interest

The authors declare no conflicts of interest.

## Supporting information




**Supporting File 1**: adma74516‐sup‐0001‐SuppMat.docx.

**Supporting File 2**: adma74516‐sup‐0002‐VideoS1.avi.

**Supporting File 3**: adma74516‐sup‐0003‐VideoS2.avi.

## Data Availability

The data that supports the findings of this study are available in the supplementary material of this article.
