# Supplementary material for: Programmable Functional Silicification of DNA Origami Nanostructures
Source: Adv Mater. 2026 Aug 5;38(56):e74516. doi: 10.1002/adma.74516 (PMC13648919; doi:10.1002/adma.74516)
Supplement: Supplementary file 1 [file ADMA-38-e74516-s003.docx]

**Supplementary Information for**

**Programmable Functional Silicification of DNA Origami Nanostructures**

Anna V. Baptist^1,2^, Lasse Guericke^1,2^, Philipp Mauker^3^, Oliver Thorn-Seshold^3^, Amelie Heuer-Jungemann^1^*

1. Faculty of Chemistry and Chemical Biology, TU Dortmund and Research Center One Health Ruhr, Otto-Hahn-Str. 6, 44227 Dortmund, Germany

2. Max Planck Institute of Biochemistry, Am Klopferspitz 18, 82152 Martinsried, and Center for Nanoscience, LMU Munich, Germany

3. Faculty of Chemistry and Food Chemistry, Dresden University of Technology, Bergstrasse 66, 01069 Dresden, Germany

* Corresponding Author

E-Mail: amelie.heuer-jungemann@tu-dortmund.de

**Figure S1: NMR spectra of the fluorescent silane**

**Figure S2: caDNAno design of the octahedral DNA origami monomers**

**Figure S3: Control experiments of unspecific binding of the fluorescent silane or the fluorescein molecules**

**Figure S4: Quantitative analysis of Figure 2b showing fluorescently silicified DNA origami after stability testing**

**Figure S5: Additional microscopy images of fluorescently silicified DNA origami crystals**

**Figure S6: Stability of fluorescently silicified DNA origami crystals after heat test, chelation test and after incubation in cell culture medium**

**Figure S7: Different time points of fluorescent silica addition to DNA origami crystals**

**Figure S8: Stability of bare, silicified and fluorescently silicified DNA origami nanostructures in cell culture medium**

**Figure S9: Stability of fluorescently silicified DNA origami octahedra after only a short incubation with the fluorescent silane during silicification**

**Figure S10: Additional data on the cellular uptake of fluorescently silicified DNA origami into HeLa cells**

**Figure S11: Colocalization analysis (fluorescein)**

**Figure S12: Cellular uptake of pure fluorescent silane or fluorescent silica clusters (without DNA origami)**

**Figure S13: Cell viability after incubation for 24 h with different silicified origami samples**

**Figure S14: Additional data pointing at a lower reactivity of BTDS compared to TEOS**

**Figure S15: Quantitative analysis of Figure 5d showing BTDS-silicified origami after DNase I treatment**

**Figure S16: Effect of GSH treatment on bare DNA origami octahedra**

**Figure S17: EDX analysis of BTDS-silicified crystals before and after treatment with GSH**

**Figure S18: CellProfiler pipeline for Cy5- and fluorescein-foci detection**

**References**

**Figure S1:** NMR spectra of the two variants of the fluorescent silica precursor (upper images: fluorescein-5-silane (S01), lower images: fluorescein-6-silane (S02)).

**Figure S2**: Scaffold routing (blue) and staple strands (black: core staples, purple: sticky ends, magenta: internal Cy5 modifications, light blue: internal fluorescein (FITC) modifications) of the DNA origami octahedra consisting of six helix bundles (6HBs). This design was created with the help of the software TALOS^1^ and caDNAno^2^. It should be noted that the positioning of the internal fluorescent modifications within the DNA origami could potentially have a significant influence on the timepoint when fluorophores are detached from the nanostructure. Here, the locations of the fluorophore-carrying staple strands at the outer parts of the 6HBs were selected to showcase a rapid loss of signal overlap.

**Figure S3**: Different control experiments to test for unspecific binding of the fluorescent silane to either the bare DNA origami or of fluorescein itself to the silica shell. (a) AGE showing the effect of the addition of the fluorescent silane to bare DNA origami octahedra with a subsequent overnight incubation (lane on the right). The band for the DNA origami nanostructures does not exhibit a fluorescent signal in the fluorescein channel, confirming that there is no noticeable unspecific binding of the fluorescent silane to the bare DNA origami. (b) AGE for control samples that were silicified (standard silicification) and then incubated for 1 d or 4 d, respectively, with fluorescein sodium salt at a similar amount compared to the fluorescent silane. Also here, no fluorescent signal in the lanes for these control samples can be observed, indicating that the fluorescein molecule alone cannot attach to the silica coating of the nanostructures.

**Figure S4:** Analysis of AGE after stability testing of fluorescently silicified DNA origami as depicted in **Figure 2b**. Shown is the fraction of the (integrated) intensity of the main band in relation to the main band + fast running degradation products (mean and its standard error). Bare and fluorescently silicified samples were compared before and after heat test and chelation test. The evaluation was done on the Cy5 channel.

**Figure S5:** Additional data for fluorescently silicified DNA origami crystals. (a) Confocal microscopy images of crystals assembled from fluorescently silicified octahedral monomers. (b) Confocal microscopy images of crystals that were silicified post-assembly. (c) TEM micrograph of fluorescently silicified crystals with a thick silica coating. Scale bars: 20 µm (a), (b) and 2 µm (c).

**Figure S6**: Stability tests for the fluorescently silicified DNA origami crystals. As expected, the fluorescently silicified crystals with their comparatively thick silica deposition can withstand the exposure to elevated temperatures (a, heat test) and low salt conditions (b, chelation test), while bare crystals fall apart. The stability of the crystals upon incubation in 1× TAE buffer (c) and DMEM with 10% FBS (d) was further monitored with the help of confocal microscopy. Even after several hours of exposure to detrimental environmental cues, the crystals still exhibit a strong fluorescent signal in the fluorescein channel. Scale bars: 1 µm (a), (b) and 20 µm (c), (d).

**Figure S7**: Confocal microscopy images for silicified crystals assembled from octahedral DNA origami monomers with different time points of fluorescent silane addition. Left columns: single images, right columns: 3D projections of z-stacks. When the fluorescent silane is added only 2 h after the start of the silicification, the fluorescent silica does not reach the center of the cubic crystals, but appears to be forming silica clusters on the outside of the structures. For the fluorescent silica addition after 4 h, there is still the formation of silica clusters around the crystals visible, even though there is also a (weak) fluorescent signal from the crystal centers. More homogeneous results can be achieved with an even later addition of the fluorescein-modified reagent, as it can be seen from the results in two lower rows. Scale bars: 20 µm.

**Figure S8**: AGE for bare, silicified and fluorescently silicified DNA origami samples after the incubation in DMEM with a final concentration of 10% FBS for up to 24 h. (i) The bare octahedron with both Cy5 and fluorescein internal modifications show clear signs of disassembly. Both the Cy5 and the fluorescein signal decrease rapidly (within the first 2 h) and a band that can probably attributed to the degradation of octahedron into 6HBs appears above the staple pools. (ii) In the silicified sample, there is still colocalization of the signals from the internal fluorophores even after 24 h and there are no additional bands appearing below the DNA origami bands, showing that the silica shell significantly enhances the stability of the nanostructures. (iii) Also, the fluorescently silicified octahedra appear to be stable and show a substantial overlap of the Cy5 and the fluorescein signal from the silica shell after 24 h in the cell culture medium. However, the fluorescein signal is decreasing over time, indicating that still some part of the originally bound fluorescent silane is detached from the nanostructures with increasing incubation time.

**Figure S9**: Behavior of silicified DNA origami octahedra after only one overnight incubation with F3EOS. (a) AGE showing the strong fluorescent signal in the DNA origami band for the fluorescently silicified octahedra. The control samples, where only methanol instead of TMAPS and TEOS was added during the first silicification round, do not exhibit a signal in the fluorescein channel and behave similarly to the bare and silicified (TEOS only) samples with internal Cy5 modifications. The fluorescently silicified octahedra exhibit a good stability upon exposure to heat (60 °C for 30 min). (b) AGE showing fluorescently silicified DNA origami samples after incubation in DMEM with a final concentration of 10% FBS for up to 24 h. Strikingly, the signal in the fluorescein channel decreases strongly over time while the Cy5 signal stays in a similar range, suggesting that the outer fluorescent silica layer is lost during incubation in the cell culture medium.

**Figure S10**: Additional data for the cellular uptake of fluorescently silicified DNA origami octahedra after 2 h, 6 h and 24 h. The control sample (fourth row, buffer only) shows only minor autofluorescence. Scale bars: 10 µm, insets: 5 µm.

**Figure S11:** Cellular uptake of bare (6 h), silicified and fluorescently silicified (both 24 h) DNA origami nanostructures. For each detected fluorescein focus at the respective time point, the measured Cy5 and fluorescein intensity is plotted below.

**Figure S12:** Control experiments for the uptake of the fluorescent silica precursor without DNA origami. The control sample with buffer only (left column) and the sample with fluorescent silane taken directly from the stock solution (middle column) do not exhibit a significant fluorescent signal. Contrary to that, the sample containing TMAPS, TEOS and fluorescent silane after incubation in buffer for 4 d and subsequent purification (right column) shows bright fluorescent spots, confirming that silica clusters with fluorescent silica can form during the silicification procedure and are not entirely removed during the purification steps. Scale bars: 10 µm, insets: 5 µm.

**Figure S13**: Cell viability determined using the cell counting kit 8 assay after incubation for 24 h with 5 nM bare DNA origami and after standard silicification (TEOS), fluorescent silicification (TEOS and F3EOS), and BTDS. The samples are compared to a buffer-only control and normalized to a cells-only control. For each sample all individual values are shown as well as the mean and its standard error (n ≥ 3).

**Figure S14:** (a) Octahedral DNA origami monomers after a ‘standard’ silicification procedure with BTDS instead of TEOS and only 6 h of incubation with the silica reagents. The resulting structures do not exhibit sufficient stability against chelation (b), indicating the need for a prolonged duration of the silicification procedure. Scale bars: 100 nm. (c) TEM micrographs showing cubic crystals that were silicified post-assembly with a mixture of BTDS and TEOS in a 5:1 ratio. The crystals appear to have a thick silica coating. (d) TEM images of such crystals after incubation with GSH. The silica coating does not exhibit any signs of disruption or dissolution, suggesting that the silica layer consists predominantly of TEOS that resists the GSH treatment. Scale bars: 1 µm.

**Figure S15:** Analysis of AGE after DNase I treatment of BTDS-silicified origami as shown in **Figure 5d**. Shown is the integrated intensity of the main band in relation to the 0 h control after 2 h, 6 h and 24 h of treatment. Bare octahedra are compared to BTDS-silicified DNA origami before and after a GSH-treatment. The curves show an exponential fit through the four points of each sample. Minor variations in the amount of loaded sample can cause slightly increased band intensities, as seen in the case of the BTDS-silicified sample.

**Figure S16**: Effect of GSH on octahedral DNA origami monomers. (a) Agarose gel showing bare octahedra that were incubated in 10 mM GSH for 3 h, followed by a purification step. There is no noticeable difference between the untreated and the GSH-treated DNA origami in the agarose gel. (b) In TEM images, there is no visible effect of the treatment with GSH on the structures of the octahedra. Scale bar: 100 nm. (c) However, after the exposure to GSH, the bare octahedra are seemingly digested even faster in the presence of DNase I. While the untreated bare octahedra are fully degraded only after 24 h, there is no DNA origami band visible already after 2 h.

**Figure S17:** EDX analysis of BTDS-silicified crystals before and after treatment with GSH. (a) representative image of a BTDS-silicified crystal. The highlighted area indicates the area used for EDX analysis. (b) Analysis of EDX data showing the ratio of Si to P. For each sample, the data was acquired by integrating the spectral signal over three different crystals, averaging the results and calculating the standard error of the mean (n = 3).

**Figure S18:** CellProfiler pipeline used to detect, filter and measure Cy5- and fluorescein-foci for colocalization analysis. Each used module is shown individually with its name and all non-default settings.

**References**

(1) Jun, H.; Shepherd, T. R.; Zhang, K.; Bricker, W. P.; Li, S.; Chiu, W.; Bathe, M. Automated Sequence Design of 3D Polyhedral Wireframe DNA Origami with Honeycomb Edges. *ACS Nano* **2019**, *13* (2), 2083–2093. DOI: 10.1021/acsnano.8b08671.

(2) Douglas, S. M.; Marblestone, A. H.; Teerapittayanon, S.; Vazquez, A.; Church, G. M.; Shih, W. M. Rapid prototyping of 3D DNA-origami shapes with caDNAno. *Nucleic Acids Research* **2009**, *37* (15), 5001–5006. DOI: 10.1093/nar/gkp436 (accessed 10/4/2025).
